# Lower Limb Lymphedema Awareness among Gynecological Cancer Patients: An International Survey Supported by the European Network of Gynecological Cancer Advocacy Groups (ENGAGe) Group

**DOI:** 10.3390/cancers16081544

**Published:** 2024-04-18

**Authors:** Dimitrios Haidopoulos, Vasilios Pergialiotis, Maria Papageorgiou, Michael J. Halaska, Katerina Maxova, Elena Ulrich, Ignacio Zapardiel, Alexandros Rodolakis, Murat Gultekin, Christina Fotopoulou

**Affiliations:** 1 First Department of Obstetrics and Gynaecology, Alexandra Hospital, National and Kapodistrian University of Athens, 2 Lour Street, 11522 Athens, Greece; dchaidop@med.uoa.gr (D.H.); arodolak@med.uoa.gr (A.R.); 2 "Erifyle" K.E.F.I. Gynecological Cancer Advocacy Group, 11526, Athens, Greece; papageorgioumaria@hotmail.com; 3 Department of Obstetrics and Gynaecology, Third Faculty of Medicine, Charles University, Faculty Hospital Kralovske Vinohrady, 11000 Prague, Czech Republic; michael.halaska@lf3.cuni.cz (M.J.H.); katerina.maxova@lf3.cuni.cz (K.M.); 4 N.N.Petrov Research Institute of Oncology, 197758 St. Petersburg, Russia; scriptamanem@yahoo.com; 5 Gynecologic Oncology Unit, La Paz University Hospital-IdiPAZ, 28046 Madrid, Spain; 6 Department of Obstetrics and Gynaecology, Hacettepe University Faculty of Medicine, Ankara 06230, Turkey; mrtgultekin@yahoo.com; 7 Department of Surgery and Cancer, Gynaecologic Oncology, Imperial College London, London W12 0HS, UK; c.fotopoulou@imperial.ac.uk

**Keywords:** lymphedema, gynecologic oncology, cancer, patient awareness, electronic survey

## Abstract

**Simple Summary:**

Patient awareness of postoperative lymphedema has been poorly explored in gynecologic oncology. While current research has extensively focused on the impact of pelvic and paraortic lymphadenectomy on patient survival, it remains relatively unknown how much of this information is actually discussed with patients that undergo surgery or radiation therapy for the treatment of their disease. In the present study we present information relevant to lymphedema awareness provided by 386 gynecological cancer patients that were treated for gynecologic malignancy. A significant lack of appropriate counseling was noted that seems to be driven by lack of appropriate physician training. This information calls for further research that will help improve patient counseling and pre-treatment decision making.

**Abstract:**

Introduction: Patient awareness of postoperative lymphedema in the field of gynecologic oncology has been poorly documented in the international literature. We wished to capture and document the awareness among gynecological cancer survivors about postoperative lymphedema, including aspects such as the adequacy of perioperative counseling, management, and quality of life. Methods: A web-based survey comprising 25 multiple-choice questions was distributed to gynecological cancer advocacy groups within the European Network of Gynecological Cancer Advocacy Groups (ENGAGe) group. The survey was validated in a pilot group of gynecological patients prior to distribution. Results: Overall, 386 women from 20 countries completed the questionnaire. Only half of the patients (*n* = 211) knew what lymphedema is, whereas 52% of the respondents stated that they were never informed at their pre-operative assessment about the potential risk of developing lymphedema. Fifty-three percent of those women who were informed about the risk and management of lymphedema received information through self-initiative, connecting mainly with patient groups or online. Approximately 84% of patients with lymphedema reported that they informed their doctor about their symptoms. Ninety-four patients (55.3%, which is not 55% of the 386) were treated for lymphedema. Forty-five women out of 136 reported that lymphedema significantly affected their everyday lives. Discussion: We report a large lack of awareness and a significant gap of knowledge about the risks and treatment options related to postoperative lymphedema among gynecological cancer survivors. Institutional practice routines and awareness among professionals need to be urgently recalled and adapted to adequately inform and support gynecological cancer patients.

## 1. Introduction

Postoperative lymphedema following gynecologic cancer surgery is a common side effect of treatment, with a cumulative prevalence ranging between 7 and 35% [1]. Endometrial, vulvar, and cervical cancers belong to the tumor types with the highest lymphedema prevalence, reaching up to 80%, respectively [2,3,4]. Patients treated for ovarian cancer have 5–20% lower rates, especially after the change of guidelines around systematic pelvic and paraaortic lymphadenectomy dictated by the LION study [5]. There is a known cumulative effect and risk of lymphedema with time [6]. Several factors seem to contribute to the occurrence, extent, and severity of lymphedema, with obesity, the radicality of lymphadenectomy, and concurrent radiotherapy as the major risk factors. The adoption of precautionary measures, such as surgical ligation of lymphatic vessels at the lymphatic tissue dissection, has been reported to be preventative, even though larger prospective randomized studies are lacking [7,8,9,10,11].

Lymphedema significantly affects the quality of life of the affected patients in terms of impaired physical functioning, bodily pain, limitations of daily activity, including physical and emotional health problems, alterations of their social functioning, and, in severe cases, mental health problems [12]. To date, the number of studies referring to the long-term impact of lymphedema on the quality of life of gynecological cancer patients is extremely limited, although it seems that the topic is of particular importance [13,14]. The importance of proactive strategies, proper patient education, and physiotherapy protocols has been extensively reviewed in cancer patients [15,16,17], although data on gynecological cancer is still missing. Poor access to appropriate treatment options to alleviate symptoms significantly affects the quality of life of patients, with the majority of patients with financial restraints reporting lower global health status scores [18]. Still, large-scale patient reported outcomes related to lower extremity lymphedema due to gynecological oncological treatment are lacking, and research is still quite heterogeneous [19]. Especially since early identification of lymphedema has been shown to limit associated morbidity, awareness and the closure of knowledge gaps not just among the affected patients but also among the treating clinicians are of crucial importance [20]. The purpose of this survey was to explore patient awareness concerning the potential occurrence of lymphedema following surgery for gynecological cancer as well as knowledge related to access to care, long-term impact, and quality of life. 

## 2. Materials and Methods

An international consortium of experts in gynecological oncological surgery designed a web-based survey comprising 25 multiple-choice questions that targeted patients` perceptions concerning: (i) their preoperative education about the possibility of developing lymphedema; (ii) knowledge of lymphedema symptoms; (iii) physician response to their symptomatology; and (iv) treatment alternatives for lymphedema prevention and treatment; as well as one open question. The survey was validated with a pilot group of patients before being launched. The survey was approved by the Institutional Review Board of the First Department of Obstetrics and Gynecology of the National and Kapodistrian University of Athens (No. 709/2020). The survey was circulated via the administration of an anonymous, non-validated, commercially available online survey (Survey Monkey™). The email inviting participation was distributed by the Erifyli group of gynecological oncology cancer patients, which is a sub-division of the Association of Cancer Patients of Athens and an official partner of the European Network of Gynecological Cancer Advocacy Groups (ENGAGe) group. The recipients of the email were all groups that participate in ENGAGe and were invited to distribute the questionnaire via email to their members. Additionally, the link to the questionnaire was also distributed by the official social media pages of ENGAGe. The full list of questions is shown in Table 1 and Table 2. All data were collected, analyzed, and extracted using the SurveyMonkey™ platform. Descriptive statistical analysis was performed by the platform, and percentages were calculated taking into account the number of responses to each individual question. 

## 3. Results

Overall, 386 women from 20 countries responded to the invitation. The distribution according to the countries with the highest participation was as follows: United Kingdom (n = 68, 17.6%) Greece (n = 50, 12.9%), The Netherlands (n = 40, 10.3%) Czech Republic (n = 33, 8.5%), Turkey (n = 32, 8.2%), Sweden (n = 31, 8.0%), and Spain (n = 30, 7.7%). Other countries with smaller participation were Finland, Hungary, Denmark, Russia, Poland, Ireland, Italy, Portugal, Turkey, Georgia, Germany, Romania, Belgium, and France. Endometrial (n = 96, 24.8%), ovarian (n = 87, 22.5), and cervical cancer (n = 159, 28.2%) constituted the majority of cancer types, with only 16 women having vulvar cancer and 8 women having vaginal cancer. 

Seventy-six percent of all patients (n = 293) had undergone surgical resection. External beam radiotherapy was used as monotherapy or combination treatment in 118 women (30.5%). Sixteen percent of patients received brachytherapy, and 40.4% of patients received chemotherapy. Three hundred and eighteen women stated that they had undergone some type of lymphadenectomy (complete or partial/sentinel lymph node), whereas 10 women did not know whether any lymphadenectomy was performed during their operation or not. 

**Figure 1:**
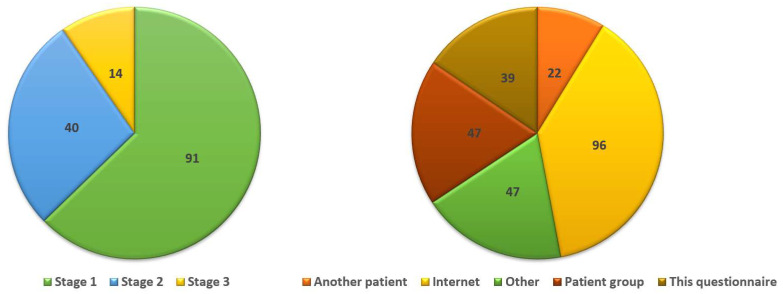
The percentage of women with signs of lymphedema assigned to its various stages of severity (**left**) and the percentage of women who received information from sources other than their physician.

Overall, 211/386 women (55%) knew what lymphedema is; however, 52% of respondents (126/243) stated that they were not informed during their pre-operative assessment of the risk of lymphedema. Twelve patients stated that they received information from their doctor only when they specifically asked about it. Seventy-eight women stated that they were not instructed to evaluate for signs of lymphedema, whereas 78/142 women (55%) stated that they were not informed about available protective measures. Approximately 52.5% (155/295) of women indicated that any information they received about lymphedema they had to search themselves online or from patient support groups (Figure 1).

**Figure 2:**
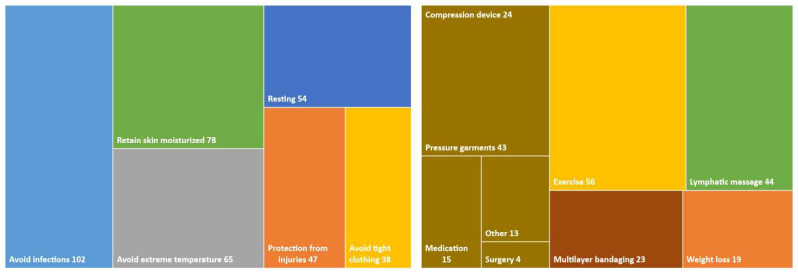
Hierarchy chart of treatments that were used by patients that suffered from lymphedema.

Of the 170 women that had signs of lymphedema (Figure 1), 103 informed their doctor about it (60.5%). One hundred and ten women received treatment (28.5% of the whole cohort). It should be emphasized that 21/170 women reported that they sought help on their own, as their physician did not refer them for treatment (12.3%). Treatment alternatives varied considerably among respondents (Figure 2), with the vast majority reporting that they were instructed to exercise, perform lymphatic massage therapy, and use compression stockings or other forms of pressure garments. 

One hundred and thirty-six women provided information about their quality of life on a scale of one to five. Of those, 45 women reported that lymphedema affected their everyday lives significantly (scale 4, 5), whereas 35 women reported that it did not affect their quality of life (scale 1). The perception of women concerning the coverage of costs from their national health system was investigated, and among the 201 respondents, 72 women (38.3%) reported that they did not know the answer to this question, 67 women responded that it was covered (33.3%), and 62 women mentioned that the cost was not covered (30.8%). Forty-seven out of 140 women (33.8%) responded that even if their national health plan/system would not cover the costs, they would still seek professional help, while 60 women (42.8%) indicated they would not do that.

Ninety-seven women provided comments on the open question. Seventy-eight women wished to complain about: inadequate preoperative information about the side effects of lymphadenectomy so that they felt they did not have the option to choose among other treatment alternatives that could decrease the chances of developing lymphedema (n = 53); their physician was not familiar with the management of lymphedema and, therefore, could not inform them adequately on treatment modalities (n = 25). 

## 4. Discussion

This is, to our knowledge, the largest-scale study of its kind that captures gynecological cancer patients’ perceptions about aspects of lymphedema arising from their anticancer treatment. We could demonstrate that there is an overall poor awareness of lymphedema, with a clear knowledge and information gap about the associated risks and treatment options, as well as inadequate access to care. Most women felt the need to seek help outside of their medical treating team due to a lack of feedback, information flow, or appropriate acting from their side. Parallel to that, we also showed a significant impact on patients QoL arising from the sequalae of the lymphedema, with the rather alarming finding that a significant proportion of these women felt that they were not adequately included in the decision processes concerning the optimal management of their own health and that they would/could have decided otherwise if they had all appropriate information about the risks and benefits of all available treatment options.

The findings of our study are very valuable in a research landscape focusing mainly on breast cancer patients, with rather scarce data about lower limb lymphedema [21]. Previous evidence has shown that breast cancer patients appear to be better informed, compared to gynecological cancer patients (25% vs. 16.8%); however, in both cases, preoperative counseling was believed to be inferior to what patients would have expected as the optimal standard. It also appears to be a lack of accurate information flow. Characteristically, Choi et al. reported that the majority (83%) of breast cancer patients had not realized that lymphedema is not completely curable in its more severe forms, while approximately 21% of them believed that there was no need to seek treatment [22]. The importance of awareness and patient education and coaching has been addressed in various other studies [21,23], with evidence clearly suggesting that adequate preoperative information about measures to counteract against lymphedema can actually result in less severe forms of lymphedema with less impact on patients’ QoL but also lower rates of associated negative symptoms such as skin necrosis and inflammation [24,25].

Several factors may affect the course and long-term impact of lymphedema, including early recognition but also patient-related aspects such as understanding the seriousness of the condition and the barriers to self-management. Ostby et al. have already, in that regard, defined specific patient profiles, including several physiological, psychological, and psychosocial factors, to be significantly associated with poorer management of lymphedema [26]. Equally important seems to be the level of education, training, and awareness of the physicians, as a large proportion of patients feel forced to seek help outside of their treating team and “selfmanage” their condition. Therefore, it is important that the gynecological oncology community recognize these gaps in order to develop personalized and symptom-oriented patient care in a more holistic and longer-term approach to cover patients’ needs. 

Early diagnosis of lymphedema that occurs following pelvic and paraortic lymphadenectomy is of particular importance, and available tools that screen for early detection of its occurrence seem to have a high sensitivity that exceeds 90% but a very low specificity that reaches only 50% [27]. Considering this, it becomes clear that more research is needed to construct diagnostic instruments with improved accuracy and establish a therapeutic plan before this complication progresses. 

Considering the results of our questionnaire, it seems that physicians are not familiar with the treatment options against lymphedema, a parameter that seems to be crucial, as the early detection of the condition does not suffice. Letting aside the preventive strategies against the formation of lymphedema, which include regular use of compression stockings and avoidance of excessive weight gain, gynecologic oncologists should become familiar with the surgical options that help alleviate symptoms and decrease severe lymphedema. Lymph node transplantation has been extensively studied in breast cancer and seems to help reduce the extent of this complication by approximately 40% [28]. Relevant information for gynecological cancer patients is missing; however, one may assume that vascularized lymph node transfer can also be considered in these cases. Lymphovenous bypass is another alternative that is based on indocyanine lymphangiography and aims at anastomosing the defective lymphatic channel with a recipient vein [29]. Low-level laser therapy has also been tested in breast cancer patients, with significant results in the reduction of lymphedema as well as accompanying pain [30].

Considering the significant symptomatology of severe lymphedema and the constraints of daily activities, psychological assessment seems to be crucial in order to help patients cope with the symptoms and improve their quality of life. Several studies have shown that lymphedema has a direct impact on patient psychology, with feelings that include negative self-identity, emotional disturbance, psychological distress, perceived diminished sexuality, social isolation, perceived social abandonment, public insensitivity, and a non-supportive work environment [31]. Considering the significant symptomatology of severe lymphedema and the constraints in daily activities, psychological assessment and early involvement seem to be crucial in order to help patients cope with the symptoms and improve their quality of life.

### 4.1. Strengths and Limitations

To our knowledge, this is one of the few studies that focused on the assessment of preoperative patient awareness of the potential occurrence of lymphedema and postoperative healthcare practices among gynecological cancer patients. The present study was distributed by a recognized society (ENGAGe), therefore ensuring the participation of patients with gynecological cancer. Nevertheless, our study is not without limitations, as the number of respondents corresponded only to a fraction of the actual number of cancer patients from the countries that participated in the survey. This can be partly attributed to the fact that the questionnaire was disseminated in English only, therefore making the access limited to patients who have the ability to communicate in English. This results in significant language bias and can also be linked to socioeconomic bias, considering that most patients who can interpret an English-language questionnaire most likely refer to westernized populations. The impact of lymphedema in more diverse populations could, therefore, help retrieve more information from patients with different cultural beliefs and sanitary habits. In our study, we also did not evaluate the potential help that the patients may have received from relatives to help fill out the questionnaire, an aspect that requires future investigation. Lastly, considering that the sample size of our study was not pre-standardized and the lack of triangulation of qualitative responses, it seems reasonable to suggest the conduct of larger studies that will help establish robust information in this important field.

### 4.2. Implications for Current Clinical Practice and Future Research

Our study has unmasked and set in plain sight the significant gaps and failures of our gynecologic oncology community to adequately address the significant problem of lower limb lymphedema in gynecological cancer patients and survivors. We have demonstrated that patients feel largely helpless and unsupported, and most importantly, that they have not had adequate information to be able to make an informed decision about their treatment options. This study is an appeal to increase awareness among healthcare professionals within the gynecologic oncology community to inform the patients early enough about the risks of lymphedema from gynecological cancer treatment so that patients not only feel more involved in the decision-making processes and are prepared for what is to come, but also to be able to counteract from the early onset and minimize damage. We need to demand as healthcare professionals that the national healthcare systems cover the financial cost of lymphedema management and have well-defined referral pathways for the affected patients without long waiting lists that will force the patients to have to self-finance their treatment, contributing to the immense financial toxicity that any cancer patient must anyway endure. 

Internationally leading societies and stakeholders need to create robust mechanisms and guidelines to address iatrogenic lymphedema, but systematic prospective studies are also warranted to capture the full extent of the problem as per tumor type and treatment modality in order to develop counteracting management. 

## Figures and Tables

**Table 1:** Lymphedema awareness questions among gynecological cancer survivors.

**Table 1. Lymphedema awareness**
**Country**
**Age**
**Type of gynecological cancer** Endometrial cancer Ovarian cancer Cervical cancer Vulvar cancer Other forms
**Year of diagnosis**
**Treatment** Surgery External beam radiotherapy Brachytherapy Chemotherapy
**If you had surgery were lymph nodes removed?**
**If you had lymph nodes removed, do you know how many? (Enter a number)**
**Do you know what lymphedema is?**
**Do you know if you have lymphedema?**
**Were you informed by your doctor about the possibility of lymphedema prior to treatment?**
**Were you informed about lymphedema by someone other than your doctor? Check all that apply.** Patient group Another patient Internet This questionnaire Other
**Do you know the stages of lymphedema?**
**Does any of the following applies to you?** I have had lymph nodes removed and/or had radiotherapy but there is no swelling (edema) (stage 0). There is swelling (edema) present which does not return to original place when pressed down. It goes away with limb elevation or rest (stage 1). There is swelling (edema) that does not resolve after limb elevation and skin fibrosis (thickening of the skin) develops (stage 2). There is swelling (oedema) that does not go away. Skin fibrosis is permanent and there are also cysts and ulcers present (stage 3). None of the above applies (You do not need to continue with the questionnaire).

**Table 2:** Awareness of treatment alternatives for lymphedema among gynecological cancer survivors.

**Table 2. Treatment awareness and quality of life assessment**
**If you have lymphedema (swollen legs) did you notify your doctor?**
**Did your doctor refer you to a medical expert?**
**Where did you seek treatment?** Physical therapist Vascular surgeon Angiologist (circulatory system doctor) I did not seek help Other (please specify)
**Was the medical expert part of your medical group?**
**Do you know how you can be diagnosed? Check all that apply.** Clinical examination Ultrasound CT scan Lymphoscintigraphy
**Were you informed on how to protect yourself from lymphedema? Check all that apply.** No Resting Protect yourself from injuries Avoid very high or very low temperatures Do not wear tight clothing Keep your skin and nails clean to avoid infections Keep your skin moisturized
**If you have lymphedema, what kind of treatment did you receive? Check all that apply.** None Diet/weight loss Exercise (walking/swimming) Lymphatic massage therapy Multilayer bandaging Compression device Pressure garments Medical therapy Surgery Laser therapy Other (please specify)
**How much does lymphedema affect your everyday life? 1 = does not affect much; 5 = affects very much.**
**If you have lymphedema, describe in a few words how it feels?**
**If it is not covered, does the cost of treatment prevent you from seeking it?**
**Is the treatment for lymphedema covered by your National Health System?**
**Do you have any additional comments on this topic? (open-ended question)**

## Data Availability

Data will become available upon reasonable request.

## References

[1] Keast D.H., Moffatt C., Janmohammad A. (2019). Lymphedema Impact and Prevalence International Study: The Canadian Data. Lymphat. Res. Biol..

[2] Huang J., Yu N., Wang X., Long X. (2017). Incidence of lower limb lymphedema after vulvar cancer: A systematic review and meta-analysis. Medicine.

[3] Yost K.J., Cheville A.L., Al-Hilli M.M., Mariani A., Barrette B.A., McGree M.E., Weaver A.L., Dowdy S.C. (2014). Lymphedema after surgery for endometrial cancer: Prevalence, risk factors, and quality of life. Obstet. Gynecol..

[4] Bona A.F., Ferreira K.R., Carvalho R.B.M., Thuler L.C.S., Bergmann A. (2020). Incidence, prevalence, and factors associated with lymphedema after treatment for cervical cancer: A systematic review. Int. J. Gynecol. Cancer.

[5] Harter P., Sehouli J., Lorusso D., Reuss A., Vergote I., Marth C., Kim J.-W., Raspagliesi F., Lampe B., Aletti G. (2019). A Randomized Trial of Lymphadenectomy in Patients with Advanced Ovarian Neoplasms. N. Engl. J. Med..

[6] Kuroda K., Yamamoto Y., Yanagisawa M., Kawata A., Akiba N., Suzuki K., Naritaka K. (2017). Risk factors and a prediction model for lower limb lymphedema following lymphadenectomy in gynecologic cancer: A hospital-based retrospective cohort study. BMC Women’s Health.

[7] Todo Y., Yamamoto R., Minobe S., Suzuki Y., Takeshi U., Nakatani M., Aoyagi Y., Ohba Y., Okamoto K., Kato H. (2010). Risk factors for postoperative lower-extremity lymphedema in endometrial cancer survivors who had treatment including lymphadenectomy. Gynecol. Oncol..

[8] Hayes S.C., Janda M., Ward L.C., Reul-Hirche H., Steele M.L., Carter J., Quinn M., Cornish B., Obermair A. (2017). Lymphedema following gynecological cancer: Results from a prospective, longitudinal cohort study on prevalence, incidence and risk factors. Gynecol. Oncol..

[9] Iyigun Z.E., Duymaz T., Ilgun A.S., Alco G., Ordu C., Sarsenov D., Aydin A.E., Celebi F.E., Izci F., Eralp Y. (2018). Preoperative Lymphedema-Related Risk Factors in Early-Stage Breast Cancer. Lymphat. Res. Biol..

[10] Prodromidou A., Iavazzo C., Fotiou A., Psomiadou V., Drakou M., Vorgias G., Kalinoglou N. (2019). The application of fibrin sealant for the prevention of lymphocele after lymphadenectomy in patients with gynecological malignancies: A systematic review and meta-analysis of randomized controlled trials. Gynecol. Oncol..

[11] Pergialiotis V., Kontzoglou K., Dimitroulis D., Vlachos D.E., Routsolias P., Vlachos G.D. (2015). Electrosurgical bipolar vessel sealing during axillary lymphadenectomy: A systematic review and meta-analysis. Breast Dis..

[12] Ahmed R.L., Prizment A., Lazovich D., Schmitz K.H., Folsom A.R. (2008). Lymphedema and quality of life in breast cancer survivors: The Iowa Women’s Health Study. J. Clin. Oncol. Off. J. Am. Soc. Clin. Oncol..

[13] Carter J., Huang H.Q., Armer J., Carlson J.W., Lockwood S., Nolte S., Kauderer J., Hutson A., Walker J.L., Fleury A.C. (2021). GOG 244-The Lymphedema and Gynecologic cancer (LeG) study: The impact of lower-extremity lymphedema on quality of life, psychological adjustment, physical disability, and function. Gynecol. Oncol..

[14] Koehler L., Penz L.E., John F., Stenzel A., Jewett P., Teoh D., Blaes A., Rivard C., Vogel R. (2023). Functional and psychosocial quality of life in gynecologic Cancer survivors with and without lymphedema symptoms. Gynecol. Oncol..

[15] Fu M.R., Axelrod D., Guth A.A., Cartwright F., Qiu Z., Goldberg J.D., Kim J., Scagliola J., Kleinman R., Haber J. (2014). Proactive approach to lymphedema risk reduction: A prospective study. Ann. Surg. Oncol..

[16] Du X., Li Y., Fu L., Chen H., Zhang X., Shui Y., Zhang A., Feng X., Fu M.R. (2022). Strategies in activating lymphatic system to promote lymph flow on lymphedema symptoms in breast cancer survivors: A randomized controlled trial. Front. Oncol..

[17] Lasinski B.B. (2013). Complete decongestive therapy for treatment of lymphedema. Semin. Oncol. Nurs..

[18] Kim S.I., Lim M.C., Lee J.S., Lee Y., Park K., Joo J., Seo S.-S., Kang S., Chung S.H., Park S.-Y. (2015). Impact of lower limb lymphedema on quality of life in gynecologic cancer survivors after pelvic lymph node dissection. Eur. J. Obstet. Gynecol. Reprod. Biol..

[19] Cemal Y., Jewell S., Albornoz C.R., Pusic A., Mehrara B.J. (2013). Systematic review of quality of life and patient reported outcomes in patients with oncologic related lower extremity lymphedema. Lymphat. Res. Biol..

[20] Krzywonos A., Ochałek K., Krzywonos-Zawadzka A., Pitala K. (2014). Assessment of knowledge of cancer and lymphoedema among breast cancer survivors. Menopause Rev. Przegląd Menopauzalny.

[21] Pervane Vural S., Ayhan F.F., Soran A. (2020). The Role of Patient Awareness and Knowledge in Developing Secondary Lymphedema after Breast and Gynecologic Cancer Surgery. Lymphat. Res. Biol..

[22] Choi J.K., Kim H.D., Sim Y.J., Kim G.C., Kim D.K., Yu B.C., Park S.S., Jeong H.J. (2015). A Survey of the Status of Awareness of Lymphedema in Breast Cancer Patients in Busan-Gyeongnam, Korea. Ann. Rehabil. Med..

[23] Borman P., Yaman A., Yasrebi S., Özdemir O. (2017). The Importance of Awareness and Education in Patients with Breast Cancer-Related Lymphedema. J. Cancer Educ..

[24] Bosompra K., Ashikaga T., O’Brien P.J., Nelson L., Skelly J., Beatty D.J. (2002). Knowledge about preventing and managing lymphedema: A survey of recently diagnosed and treated breast cancer patients. Patient Educ. Couns..

[25] Fu M.R., Chen C.M., Haber J., Guth A.A., Axelrod D. (2010). The effect of providing information about lymphedema on the cognitive and symptom outcomes of breast cancer survivors. Ann. Surg. Oncol..

[26] Ostby P.L., Armer J.M., Smith K., Stewart B.R. (2018). Patient Perceptions of Barriers to Self-Management of Breast Cancer-Related Lymphedema. West. J. Nurs. Res..

[27] Armbrust R., Auletta V., Cichon G., Vercellino G., Yost K., Sehouli J. (2023). Lymphedema after pelvic and para-aortic lymphadenectomy—Results of a systematic evaluation in patients with cervical and endometrial carcinoma. Arch. Gynecol. Obstet..

[28] Winters H., Tielemans H.J.P., Paulus V., Hummelink S., Slater N.J., Ulrich D.J.O. (2022). A systematic review and meta-analysis of vascularized lymph node transfer for breast cancer-related lymphedema. J. Vasc. Surg. Venous Lymphat. Disord..

[29] Chang E.I., Skoracki R.J., Chang D.W. (2018). Lymphovenous Anastomosis Bypass Surgery. Semin. Plast. Surg..

[30] Smoot B., Chiavola-Larson L., Lee J., Manibusan H., Allen D.D. (2015). Effect of low-level laser therapy on pain and swelling in women with breast cancer-related lymphedema: A systematic review and meta-analysis. J. Cancer Surviv..

[31] Fu M.R., Ridner S.H., Hu S.H., Stewart B.R., Cormier J.N., Armer J.M. (2013). Psychosocial impact of lymphedema: A systematic review of literature from 2004 to 2011. Psychooncology.

